# Association of leucine pathway metabolites with moyamoya disease and its clinical phenotypes

**DOI:** 10.3389/fneur.2026.1875390

**Published:** 2026-10-08

**Authors:** Miaoran Fu, Jixuan Xu, Tianjiao Zhang, Yueming Luo, Lijun Wang, Lulu Qi, Min Zhang, Chuming Tao

**Affiliations:** 1 Department of Neurology, The Fifth Affiliated Hospital of Zhengzhou University, Zhengzhou, China; 2 Department of Neurosurgery, Beijing Tiantan Hospital, Capital Medical University, Beijing, China; 3 Department of Neurosurgery, The Second Affiliated Hospital of Nanchang University, Nanchang, China; 4 Department of Neurology, The Second Affiliated Hospital of Nanchang University, Nanchang, China; 5 Department of Gastrointestinal & Thyroid Surgery, The Fifth Affiliated Hospital of Zhengzhou University, Zhengzhou, China; 6 Medicine School, Hubei Minzu University, Enshi, China

**Keywords:** branched-chain amino acids, leucine, metabolomics, moyamoya disease, N-isovalerylglycine

## Abstract

**Background:**

Leucine and its metabolic derivatives have emerged as promising candidates for biomarker discovery in cardio-cerebrovascular diseases. Despite this, their involvement in the pathophysiology and phenotypic expression of moyamoya disease (MMD) remains largely unexplored. This investigation therefore sought to delineate the potential links between perturbations in the leucine metabolic pathway and susceptibility to MMD.

**Methods:**

This was a cross-sectional case–control study with prospective participant enrollment (337 MMD patients; 84 age- and sex-comparable healthy controls). Serum leucine, glycine, and N-isovalerylglycine (N-IVA) were quantified via LC–MS/MS. Multivariable logistic regression and ROC analysis (including NRI and IDI) were used to assess independent associations and incremental predictive value.

**Results:**

Relative to healthy controls, patients with MMD exhibited higher serum leucine concentrations [median (IQR): 217.91 (186.70–245.32) vs. 188.05 (166.09–217.76) μmol/L, *p* < 0.001], accompanied by lower N-isovalerylglycine (N-IVA) levels (*p* = 0.004). After adjustment for clinical and laboratory variables, higher leucine levels remained independently linked to an increased probability of MMD (OR = 1.01, 95% CI: 1.00–1.02, *p* = 0.010), whereas higher N-isovalerylglycine (N-IVA) levels were associated with lower odds of MMD (per 1-SD increase, OR = 0.74, 95% CI: 0.55–1.00, *p* = 0.049). Incorporation of leucine into the baseline clinical model resulted in improved risk stratification (categorical NRI = 0.140, *p* = 0.007). In addition, the basic clinical model achieved an AUC of 0.880, which increased to 0.892 after incorporating leucine, glycine, and N-IVA, reflecting a modest but consistent increase in discrimination, and further enhanced reclassification performance (NRI: 0.193, *p* < 0.001; IDI: 0.032, *p* = 0.011). Serum leucine concentrations also showed positive correlations with inflammatory and lipid-related indicators.

**Conclusion:**

Alterations in leucine metabolism, reflected by elevated leucine and reduced N-isovalerylglycine levels, are associated with MMD and contribute additional predictive value beyond conventional clinical factors. These results indicate that alterations in leucine pathway metabolism are associated with MMD and may reflect broader systemic metabolic disturbances relevant to its pathophysiology, warranting further mechanistic investigation.

## 1 Introduction

Characterized as a chronic progressive cerebrovascular disorder, Moyamoya disease (MMD) is fundamentally defined by the progressive constriction and eventual blockage affecting the distal segments of the internal carotid arteries. This progressive arterial occlusion stimulates the development of a hazy, smoke-like collateral circulation (moyamoya vessels) on cerebral angiography (1, 2). While the contributions of genetic susceptibility, particularly the RNF213 variant, vascular inflammation, and aberrant angiogenesis has been well documented, the systemic metabolic shifts underlying MMD pathophysiology remain largely elusive (1, 3). The cerebrovascular system exhibits a high level of metabolic vulnerability (4, 5), such that dysregulated circulating metabolites may serve as potential circulating biomarkers associated with MMD (3, 6). Notably, an untargeted metabolomic profiling study in MMD identified circulating lipid metabolites, including reduced LPC 16:1, as candidate biomarkers distinguishing disease subtypes and linked to pathological angiogenesis (6), underscoring the broader relevance of circulating metabolic alterations to MMD biology.

Branched-chain amino acids (BCAAs) play essential roles in systemic energy homeostasis and have been increasingly implicated in vascular function, inflammatory regulation, and mitochondrial metabolism (7–9). Among BCAAs, leucine has been recognized as an important regulator of metabolic signaling and energy balance (10, 11). Accumulating evidence suggests that dysregulated BCAA metabolism is associated with a range of cardiovascular and cerebrovascular disorders, including atherosclerosis and ischemic stroke, possibly through oxidative stress and inflammatory pathways (12, 13). Under physiological stress or enzymatic deficiency, leucine may be diverted into an alternative detoxification pathway, resulting in the formation of N-isovalerylglycine (N-IVA) (14). While N-IVA has been established as a sensitive marker in organic acidemias (15), its relevance to moyamoya disease and its association with disease-related phenotypes remain a significant knowledge gap.

Therefore, this study intends to explore the associations between circulating leucine and its related metabolites, including glycine and N-isovalerylglycine, and moyamoya disease. This targeted focus builds on our group’s related investigation of total and individual circulating branched-chain amino acids (leucine, isoleucine, and valine) in the same prospectively enrolled population (16), which found broadly similar associations with MMD across the three amino acids; the present study therefore concentrates specifically on leucine and its downstream shunt metabolite N-IVA, a relationship not examined in that earlier work. We further examined the relationships of these metabolites with distinct clinical phenotypes of MMD, including ischemic and hemorrhagic subtypes. Additionally, the correlation between these metabolic signatures and clinical characteristics, such as Suzuki stage and laboratory parameters, was explored to better understand the potential role of leucine metabolism in the pathophysiology of MMD.

## 2 Materials and methods

### 2.1 Study design and participants

This was a cross-sectional case–control study with prospective participant enrollment of patients with moyamoya disease (MMD) treated at Beijing Tiantan Hospital from September 2020 to December 2021. The participant selection process is presented in Figure 1. MMD was diagnosed by experienced cerebrovascular specialists at our institution on the basis of digital subtraction angiography (DSA), in line with the 2012 Japanese guidelines (17), as most recently revised in the 2021 diagnostic criteria (18). Based on these criteria, all patients were diagnosed with MMD via DSA according to the radiographic detection of unilateral or bilateral steno-occlusive changes at the terminal portion of the intracranial internal carotid artery or at the proximal segments of the anterior and/or middle cerebral arteries. The requisite compensatory smoky-like vessels at the cranial base were verified through digital subtraction angiography.

**Figure 1:**
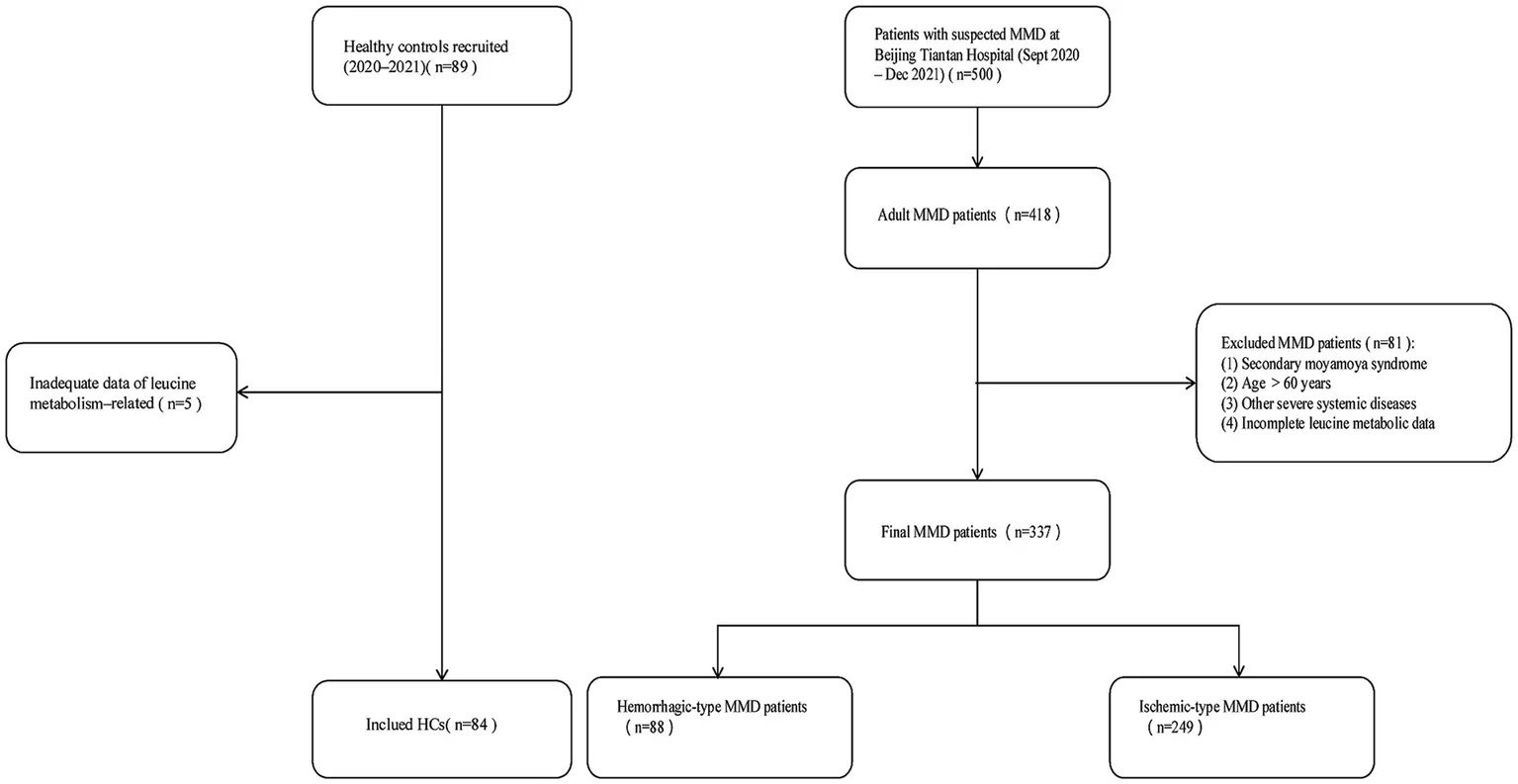
Flowchart of study participants. A total of 421 participants were analyzed, comprising 337 patients with moyamoya disease (MMD; 249 ischemic, 88 hemorrhagic) and 84 age- and sex-comparable healthy controls (HCs) recruited from the hospital health-screening department, after exclusion of patients with secondary moyamoya syndrome, age >60 years, severe systemic comorbidities, or incomplete leucine-pathway metabolite data. MMDs, moyamoya disease patients; HCs, healthy controls.

Exclusion criteria were applied to ensure study stringency: (1) secondary moyamoya syndrome caused by underlying etiologies such as atherosclerosis, autoimmune diseases, brain tumors, or cranial irradiation; (2) age older than 60 years; (3) presence of other severe systemic diseases (e.g., cancer, or end-stage renal disease); and (4) patients without complete data of leucine metabolism–related metabolites.

For the comparative analysis, we enrolled an age-comparable control group consisting of visitors at Beijing Tiantan Hospital’s health screening department, without a history of vascular risk factors or cerebrovascular disease. Every participant provided written informed consent before being recruited for the study. The study protocol received approval from the Institutional Review Board of Beijing Tiantan Hospital (Approval No. KY2022-051-02).

### 2.2 Clinical assessment and laboratory data collection

Demographic characteristics, clinical parameters, and laboratory data were obtained using standardized case report forms and electronic medical records at admission. Baseline clinical information included age, sex, heart rate, blood pressure, and body mass index (BMI). The angiographic severity of MMD was evaluated using the Suzuki stage based on digital subtraction angiography (DSA) images, as assessed by cerebrovascular specialists. MMD patients were further classified into ischemic and hemorrhagic subtypes according to the corresponding neuroimaging findings (cerebral infarction versus intracranial hemorrhage) at initial presentation; patients with nonspecific symptoms (e.g., headache, dizziness) or incidentally detected disease were classified according to the accompanying neuroimaging findings (e.g., silent infarction was classified as the ischemic subtype), ensuring complete phenotype assignment for all participants.

Routine biochemical, hematological, and metabolic assays were conducted on fasting blood samples at the clinical laboratory of Beijing Tiantan Hospital. The measured parameters encompassed: (1) hematologic markers, including white blood cell and neutrophil counts, along with platelet indices; (2) hepatic and renal function indicators such as ALT, GGT, total and indirect bilirubin, and creatinine; (3) lipid profiles comprising total cholesterol(CHO), triglycerides(TG), HDL-C, LDL-C, apolipoprotein A1(APO-A1), and apolipoprotein B(APO-B); (4) electrolytes as well as homocysteine (Hcy). For MMD patients who provided additional written consent, genetic screening for the RNF213 p. R4810K variant (rs112735431) was performed using the following primer pair: forward, 5′-GCCCTCCATTTCTAGCACAC-3′; reverse, 5′-AGCTGTGGCGAAAGCTTCTA-3′.

### 2.3 Serum sample collection and metabolite measurement

For MMD patients, fasting peripheral venous blood was collected on the morning of the second hospital day, following an overnight fast and prior to any surgical intervention, to minimize the influence of acute-phase physiological stress related to the presenting event on circulating metabolite levels; healthy controls underwent the same fasting blood collection procedure at their health-screening visit. All samples were collected in serum separation tubes. The samples were promptly centrifuged, aliquoted, and stored at −80 °C in the Central Laboratory of Beijing Tiantan Hospital within 1 h after collection. The serum metabolite data used in the present study were obtained from a larger prospectively established metabolomic database at Beijing Tiantan Hospital. The present analytic population was selected from this parent database according to the eligibility and data-completeness criteria specific to the current study and was therefore not identical to the analytic populations included in our previous reports (16, 19). The present analysis extracted and evaluated serum measurements of leucine, glycine, and N-isovalerylglycine from this parent database.

Targeted quantification of leucine, glycine, and N-isovalerylglycine (N-IVA) was performed via liquid chromatography–tandem mass spectrometry (LC–MS/MS) on an AB Sciex QTRAP 6500 platform (MetWare, Wuhan, China). Briefly, serum samples were thawed on ice and denatured with organic solvents containing internal standards; after centrifugation, supernatants were analyzed under gradient elution on a dedicated chromatographic column with electrospray ionization in multiple reaction monitoring (MRM) mode.

For quality control, pooled QC samples were prepared by mixing equal-volume aliquots from all subjects and were periodically analyzed at every ten injections to monitor analytical stability. MultiQuant software was employed for raw data processing, enabling accurate peak integration and signal verification. Throughout the study, laboratory staff conducting metabolomic quantification and data analysis were kept unaware of all participant-specific clinical and diagnostic information.

### 2.4 Statistical analysis

Before the formal analysis, the distributional properties of all continuous variables were assessed to inform appropriate summary statistics and inferential methods. Data were reported as mean ± standard deviation (SD) when approximately normally distributed, or as median with interquartile range (IQR) when skewed; categorical variables were presented as frequencies and percentages. For between-group analyses, normally distributed continuous variables were examined using Student’s t-test, whereas non-normally distributed data were analyzed with the Mann–Whitney U test. Differences in categorical variables were evaluated using either the chi-square test or Fisher’s exact test, as appropriate.

Associations between leucine pathway metabolites (leucine, glycine, and N-isovalerylglycine) and MMD were evaluated using multivariable logistic regression, with effect estimates expressed as odds ratios (ORs) and 95% confidence intervals (CIs). The concentrations of these metabolites were divided into tertiles (T1 - T3) based on their natural distribution patterns, with T1 set as the reference group. We compared the characteristics among different tertiles and assessed linear trends by regarding the tertile groups as an ordinal predictor.

The crude model refers to the unadjusted univariable association between each metabolite and MMD. Two adjusted logistic regression models were additionally constructed. Model 1 included age, sex, heart rate, systolic blood pressure (SBP), diastolic blood pressure (DBP), and body mass index (BMI). Model 2 was further adjusted for additional laboratory parameters, including white blood cell count (WBC), neutrophil count (NEUT), platelet count (PLT), gamma-glutamyl transferase (GGT), fasting glucose (Glu), creatinine (Cr), uric acid (UA), triglycerides (TG), total cholesterol (CHO), high-density lipoprotein cholesterol (HDL-C), low-density lipoprotein cholesterol (LDL-C), apolipoprotein A1 (APO-A1), apolipoprotein B (APO-B), homocysteine (Hcy), and estimated glomerular filtration rate (eGFR); these covariates were selected on the basis of their significant between-group differences in Table 1 and their established relevance as cardiometabolic confounders of amino-acid metabolism. To further address the substantial imbalance in group size and the possibility of residual confounding by cardiovascular risk-factor burden between the MMD and control groups, we additionally performed propensity-score matching on age, sex, and BMI (1:3 caliper matching, caliper = 0.2 SD of the logit propensity score) and a sensitivity analysis restricting the MMD group to patients without any of five conventional vascular risk factors (hypertension, diabetes, hyperlipidemia, smoking, alcohol use), each compared against the full control group; the results of these sensitivity analyses are reported in the Results section and Supplementary Table S4. All models developed in this study are cross-sectional diagnostic (discriminative) models distinguishing prevalent MMD cases from controls at a single time point; no prognostic model predicting future clinical events (e.g., stroke recurrence or disease progression) was developed or evaluated, given the absence of longitudinal outcome data in this study design.

**Table 1:** Baseline characteristics of participants.

Variables	HCs (*n* = 84)	MMDs (*n* = 337)	*p*-Value
Age	40.00 (30.50, 50.00)	43.00 (34.00, 50.00)	0.123
Male sex, *n* (%)	50 (59.5)	197 (58.5)	0.957
BMI, kg/m^2^	23.00 (21.00, 26.00)	25.00 (23.00, 28.00)	0.001*
Cardiovascular risk factors			
Hypertension (%)	0 (0.0)	126 (37.4)	<0.001*
Diabetes mellitus (%)	0 (0.0)	57 (16.9)	<0.001*
Hyperlipidemia (%)	0 (0.0)	54 (16.0)	<0.001*
Cigarette smoking (%)	0 (0.0)	63 (18.7)	<0.001*
Alcohol drinking (%)	0 (0.0)	41 (12.2)	0.002*
Hyperhomocysteinemia, *n* (%)	0 (0.0)	14 (4.2)	0.119
SBP, mmHg	124.00 (115.00, 130.00)	133.00 (124.00, 140.00)	<0.001*
DBP, mmHg	78.00 (73.75, 81.00)	82.00 (77.00, 89.00)	<0.001*
Heart rate, bpm	78.00 (70.00, 85.00)	78.00 (75.00, 80.00)	0.181
Hematological indices			
WBC, 109/L	6.06 (5.03, 6.89)	6.87 (5.71, 8.15)	<0.001*
NEUT, 109/L	3.45 (2.80, 4.41)	4.25 (3.42, 5.24)	<0.001*
GR, %	58.60 (54.58, 66.58)	62.70 (58.00, 68.60)	0.001*
PLT, 109/L	239.00 (202.75, 285.25)	248.00 (211.00, 286.00)	0.367
PDW, fL	16.10 (15.90, 16.33)	16.10 (15.80, 16.30)	0.238
MPV, fL	9.40 (8.70, 10.40)	9.50 (8.80, 10.20)	0.727
P-LCR, %	22.75 (17.75, 27.15)	22.60 (18.10, 27.70)	0.911
PCT, %	0.23 (0.20, 0.26)	0.23 (0.20, 0.27)	0.463
Liver function markers			
ALT, U/L	18.35 (13.38, 23.20)	21.10 (14.10, 31.00)	0.046*
TP, g/L	72.95 (70.77, 75.00)	73.50 (70.50, 76.30)	0.631
ALB, g/L	44.80 (43.50, 46.52)	45.50 (43.40, 47.40)	0.235
ALP, U/L	56.70 (49.85, 71.73)	69.70 (56.70, 82.70)	<0.001*
GGT, U/L	16.90 (12.43, 28.22)	23.40 (15.80, 34.30)	0.001*
TBIL, μmol/L	12.02 (9.74, 15.67)	11.20 (8.56, 14.17)	0.040*
IBIL, μmol/L	9.25 (7.40, 11.25)	8.00 (6.20, 10.20)	0.001*
CHE, U/L	8606.00 (7512.00, 9619.00)	9204.00 (7967.00, 10256.00)	0.010*
Myocardial/tissue enzymes			
LDH, U/L	172.20 (155.10, 187.63)	172.20 (155.80, 192.60)	0.630
CK, U/L	84.10 (62.45, 112.27)	69.10 (53.00, 97.00)	0.003*
HBDH, U/L	128.70 (114.30, 136.95)	132.60 (118.80, 145.60)	0.029*
Renal and metabolic indicators			
UA, μmol/L	310.50 (251.10, 351.80)	307.70 (254.40, 368.50)	0.657
eGFR, ml/min per 1.73 m2	124.19 (112.58, 135.56)	123.57 (115.26, 134.37)	0.997
Cr, μmol/L	57.45 (49.48, 68.12)	55.1 0 (46.40, 66.40)	0.212
Glu, mmol/L	5.04 (4.76, 5.37)	5.15 (4.76, 5.82)	0.056
CO2, mmol/L	23.00 (22.00, 24.13)	25.00 (23.30, 26.90)	<0.001*
Ca, mmol/L	2.41 (2.35, 2.48)	2.44 (2.38, 2.50)	0.007*
P, mmol/L	1.08 (0.96, 1.20)	1.13 (1.02, 1.25)	0.010*
Lipid profile			
TG, mmol/L	0.84 (0.65, 1.21)	1.20 (0.84, 1.64)	<0.001*
CHO, mmol/L	4.68 (4.16, 5.10)	4.23 (3.52, 4.88)	<0.001*
HDL, mmol/L	1.53 (1.34, 1.74)	1.30 (1.12, 1.49)	<0.001*
LDL, mmol/L	2.70 (2.26, 3.14)	2.37 (1.82, 3.01)	0.001*
APO-A1,g/L	1.40 (1.28, 1.54)	1.30 (1.15, 1.46)	<0.001*
APO-B,g/L	0.77 (0.69, 0.95)	0.82 (0.70, 0.98)	0.273
Electrolytes			
Na, mmol/L	140.05 (139.00, 141.02)	141.40 (140.00, 143.00)	<0.001*
K, mmol/L	4.11 (3.94, 4.26)	4.21 (3.99, 4.42)	0.006*
RNF213 p. R4810K (%)	0 (0.0)	60 (17.8)	<0.001*
Hcy, μmol/L	10.45 (8.65, 12.40)	12.09 (9.40, 15.64)	<0.001*
N-Isovalerylglycine, μmol/L	1.03 (0.98, 1.08)	1.00 (0.96, 1.05)	0.004*
Glycine, μmol/L	454.41 (413.88, 555.46)	457.62 (402.87, 537.56)	0.482
Leucine, μmol/L	188.05 (166.09, 217.76)	217.91 (186.70, 245.32)	<0.001*

MMD, moyamoya disease; HCs, healthy controls; BMI, body mass index; SBP, systolic blood pressure; DBP, diastolic blood pressure; WBC, white blood cell; NEUT, neutrophil; GR, granulocyte ratio; PLT, platelet; PDW, platelet distribution width; MPV, mean platelet volume; P-LCR, platelet large cell ratio; PCT, plateletcrit; ALT, alanine aminotransferase; TP, total protein; ALB, albumin; ALP, alkaline phosphatase; GGT, gamma-glutamyl transferase; TBIL, total bilirubin; IBIL, indirect bilirubin; CHE, cholinesterase; LDH, lactate dehydrogenase; CK, creatine kinase; HBDH, hydroxybutyrate dehydrogenase; UA, uric acid; eGFR, estimated glomerular filtration rate; Cr, creatinine; Glu, glucose; TG, triglycerides; CHO, total cholesterol; HDL-C, high-density lipoprotein cholesterol; LDL-C, low-density lipoprotein cholesterol; APO-A1, apolipoprotein A1; APO-B, apolipoprotein B; Hcy, homocysteine. Data are presented as median (interquartile range, IQR) for continuous variables and number (percentage) for categorical variables. *p* values were calculated using the Mann–Whitney U test for continuous variables and the chi-square test or Fisher’s exact test for categorical variables.*p < 0.05 was considered statistically significant.

Receiver operating characteristic (ROC) curve analysis was applied to assess the discriminative capacity of individual metabolites, with calculation of the area under the curve (AUC) and its 95% confidence intervals. Improvements in predictive performance beyond conventional clinical models were evaluated using changes in AUC, net reclassification improvement (NRI), and integrated discrimination improvement (IDI).

R software (version 4.5.2, macOS) was used for all analyses. All tests were two-sided, with a *p* value < 0.05 indicating statistical significance.

## 3 Results

### 3.1 Baseline characteristics and subtype comparison

A total of 421 individuals were analyzed, including 337 patients with MMD and 84 age- and sex-comparable healthy controls (HCs) (Figure 1). Baseline characteristics are summarized in Table 1. Median age was comparable between groups (*p* = 0.123), and no difference in sex distribution was observed (*p* = 0.957), confirming that age and sex distributions were comparable between groups. Of the 337 MMD patients, 300 (89.0%) had bilateral and 37 (11.0%) had unilateral disease on DSA (Ischemic subtype: 227/249 bilateral, 22/249 unilateral; Hemorrhagic subtype: 73/88 bilateral, 15/88 unilateral).

Compared with healthy controls (HCs), individuals with MMD exhibited a substantially greater prevalence of conventional cardiovascular risk factors, such as hypertension, diabetes mellitus, hyperlipidemia, as well as a history of tobacco and alcohol use (all *p* < 0.05). Regarding physiological measures, MMD patients displayed significantly elevated systolic blood pressure [133.00 (124.00, 140.00) vs. 124.00 (115.00, 130.00) mmHg; *p* < 0.001], diastolic blood pressure [82.00 (77.00, 89.00) vs. 78.00 (73.75, 81.00) mmHg; *p* < 0.001], and body mass index (BMI) [25.00 (23.00, 28.00) vs. 23.00 (21.00, 26.00) kg/m^2^; *p* = 0.001]. Laboratory analyses further indicated modestly higher hematological indices in MMD patients, although values remained largely within the normal reference range, reflected by increased white blood cell (WBC) counts, neutrophil counts, and granulocyte proportions (all *p* < 0.001). Additionally, the MMD group showed altered lipid metabolism and mildly elevated hepatobiliary enzyme levels, characterized by increased triglycerides (TG), alkaline phosphatase (ALP), gamma-glutamyl transferase (GGT), and cholinesterase (CHE), coupled with decreased total cholesterol (CHO), HDL-C, and LDL-C levels (all *p* < 0.05). Because GGT was already included as a covariate in the fully adjusted Model 2, the leucine and N-IVA associations reported under Model 2 already account for this potential hepatic confounder. Notably, serum homocysteine (Hcy) levels were also substantially higher in MMD patients than in HCs [12.09 (9.40, 15.64) vs. 10.45 (8.65, 12.40) μmol/L; *p* < 0.001].

Subgroup analysis comparing MMD subtypes with healthy controls (HCs) is presented in Table 2. Age and sex distribution were comparable among groups. Both ischemic and hemorrhagic MMD patients exhibited a greater burden of vascular risk factors than HCs. Hypertension was observed in 40% of patients with ischemic MMD and 31% of those with hemorrhagic MMD (both *p* < 0.001 vs. HCs), whereas diabetes and hyperlipidemia were more prevalent in the ischemic subtype. Additionally, both MMD subtypes exhibited higher systolic and diastolic blood pressure compared with healthy controls (all *p* < 0.05). Ischemic MMD patients demonstrated more pronounced metabolic and inflammatory abnormalities, including higher BMI (*p* < 0.001), triglyceride levels (*p* < 0.001), and neutrophil counts (*p* < 0.001), accompanied by lower HDL-C concentrations (*p* < 0.001). In contrast, hemorrhagic MMD showed relatively milder metabolic alterations, although triglyceride and HDL-C levels remained significantly different from those of HCs (*p* < 0.05).

**Table 2:** Comparison of clinical characteristics among healthy controls and patients with ischemic and hemorrhagic moyamoya disease.

Variables	HC *N* = 84	IschemicMMD *N* = 249	*p* value	HemorrhagicMMD *N* = 88	*p* value
Age, years	39.92 ± 11.79	42.46 ± 10.42	0.081	41.89 ± 10.78	0.255
Gender, female, *n* (%)	50 (60%)	138 (55%)	0.597	59 (67%)	0.387
BMI, kg/m^2^	23.76 ± 3.37	25.89 ± 4.41	<0.001*	24.34 ± 4.30	0.326
Cardiovascular risk factors					
Hypertension, *n* (%)	0 (0%)	99 (40%)	<0.001*	27 (31%)	<0.001*
Diabetes, *n* (%)	0 (0%)	53 (21%)	<0.001*	4 (4.5%)	0.141
Hyperlipidemia, *n* (%)	0 (0%)	45 (18%)	<0.001*	9 (10%)	0.008*
Smoking, *n* (%)	0 (0%)	50 (20%)	<0.001*	13 (15%)	<0.001*
Drinking, *n* (%)	0 (0%)	35 (14%)	<0.001*	6 (6.8%)	0.043*
Hyperhomocysteinemia, *n* (%)	0 (0%)	12 (4.8%)	0.087	2 (2.3%)	0.498
SBP, mmHg	122.96 ± 11.34	134.36 ± 13.53	<0.001*	129.52 ± 12.42	<0.001*
DBP, mmHg	78.01 ± 8.25	82.85 ± 9.24	<0.001*	80.74 ± 9.13	0.041*
Heart rate, bpm	78.02 ± 9.40	78.46 ± 6.76	0.697	79.34 ± 5.91	0.276
Hematological indices					
WBC, 10^9^/L	6.10 ± 1.50	7.32 ± 2.02	<0.001*	7.00 ± 2.25	0.002*
Neutrophil, 10^9^/L	3.72 ± 1.34	4.69 ± 1.83	<0.001*	4.68 ± 2.37	0.001*
GR, %	59.89 ± 8.70	62.97 ± 8.50	0.006*	64.49 ± 11.44	0.003*
Platelet, 10^9^/L	249.56 ± 60.12	257.63 ± 72.24	0.314	252.30 ± 72.30	0.787
PDW, fL	16.05 ± 0.67	16.06 ± 0.36	0.866	16.06 ± 0.47	0.928
MPV, fL	9.60 ± 1.08	9.51 ± 1.00	0.485	9.53 ± 1.11	0.648
P-LCR, %	23.51 ± 7.28	23.12 ± 6.78	0.666	23.22 ± 7.34	0.792
PCT, %	0.24 ± 0.05	0.24 ± 0.06	0.319	0.24 ± 0.06	0.983
Liver function markers					
ALT, U/L	22.18 ± 14.71	28.36 ± 24.97	0.007*	25.08 ± 21.52	0.301
Total protein, g/L	73.17 ± 3.09	72.77 ± 6.62	0.456	74.05 ± 3.96	0.103
Albumin, g/L	45.08 ± 2.24	45.22 ± 3.13	0.652	45.35 ± 2.74	0.480
ALP, U/L	61.28 ± 15.63	74.77 ± 43.91	<0.001*	74.89 ± 31.65	<0.001*
GGT, U/L	24.11 ± 21.48	32.60 ± 35.88	0.010*	24.52 ± 14.90	0.885
Total bilirubin, μmol/L	12.78 ± 4.19	12.17 ± 4.90	0.266	11.26 ± 4.84	0.029*
Indirect bilirubin, μmol/L	9.54 ± 3.19	8.63 ± 3.56	0.030*	8.03 ± 3.03	0.002*
Cholinesterase(CHE), U/L	8,639.05 ± 1,651.31	9,363.63 ± 1,709.63	<0.001*	8,924.82 ± 1,958.86	0.302
Myocardial/tissue enzymes					
LDH, U/L	172.28 ± 24.11	175.10 ± 29.33	0.382	174.69 ± 24.19	0.514
CK, U/L	92.45 ± 42.23	85.10 ± 64.34	0.234	74.37 ± 36.09	0.003*
HBDH, U/L	128.16 ± 19.41	133.66 ± 22.20	0.032*	133.72 ± 18.17	0.054
Renal and metabolic indicators					
UA, μmol/L	310.01 ± 74.11	321.36 ± 90.22	0.253	298.76 ± 86.99	0.362
eGFR, ml/min per 1.73 m2	123.92 ± 14.95	124.30 ± 15.20	0.838	124.23 ± 15.81	0.894
Creatinine, μmol/L	58.00 ± 15.31	57.35 ± 13.62	0.731	55.75 ± 13.91	0.315
Glu, mmol/L	5.10 ± 0.51	5.80 ± 1.79	<0.001*	5.15 ± 0.90	0.648
CO2, mmol/L	23.17 ± 2.33	25.04 ± 2.53	<0.001*	24.54 ± 2.32	<0.001*
Calcium, mmol/L	2.41 ± 0.09	2.44 ± 0.09	0.013*	2.44 ± 0.10	0.052
Phosphate, mmol/L	1.08 ± 0.17	1.13 ± 0.17	0.022*	1.15 ± 0.18	0.007*
Lipid profile					
Triglyceride, mmol/L	1.06 ± 0.71	1.51 ± 1.29	<0.001*	1.34 ± 0.84	0.020*
Total cholesterol, mmol/L	4.67 ± 0.73	4.13 ± 1.10	<0.001*	4.60 ± 0.97	0.552
HDL-C, mmol/L	1.54 ± 0.28	1.28 ± 0.28	<0.001*	1.39 ± 0.27	<0.001*
LDL-C, mmol/L	2.74 ± 0.67	2.31 ± 0.82	<0.001*	2.77 ± 0.89	0.744
ApoA1, g/L	1.41 ± 0.18	1.30 ± 0.25	<0.001*	1.33 ± 0.25	0.017*
ApoB, g/L	0.82 ± 0.21	0.83 ± 0.23	0.863	0.90 ± 0.25	0.021*
Electrolytes					
Sodium, mmol/L	140.30 ± 1.78	141.48 ± 2.34	<0.001*	141.46 ± 2.54	<0.001*
Potassium, mmol/L	4.11 ± 0.30	4.24 ± 0.38	0.002*	4.14 ± 0.34	0.563
RNF213 p. R4810K, n (%)	0 (0%)	44 (18%)	<0.001*	16 (18%)	<0.001*
Homocysteine, μmol/L	11.16 ± 6.44	13.80 ± 7.42	0.002*	12.96 ± 5.77	0.055
N-Isovalerylglycine, μmol/L	1.06 ± 0.15	1.02 ± 0.09	0.029*	1.02 ± 0.10	0.065
Glycine, μmol/L	480.93 ± 100.21	469.32 ± 108.64	0.370	495.61 ± 131.12	0.409
Leucine, μmol/L	193.86 ± 40.34	223.13 ± 45.43	<0.001*	210.87 ± 45.68	0.010*

HC, healthy control; MMD, moyamoya disease; BMI, body mass index; SBP, systolic blood pressure; DBP, diastolic blood pressure; WBC, white blood cell; NEUT, neutrophil; GR, granulocyte ratio; PLT, platelet; PDW, platelet distribution width; MPV, mean platelet volume; P-LCR, platelet large cell ratio; PCT, plateletcrit; ALT, alanine aminotransferase; ALP, alkaline phosphatase; GGT, gamma-glutamyl transferase; LDH, lactate dehydrogenase; CK, creatine kinase; HBDH, hydroxybutyrate dehydrogenase; UA, uric acid; eGFR, estimated glomerular filtration rate; Cr, creatinine; Glu, glucose; TG, triglycerides; HDL-C, high-density lipoprotein cholesterol; LDL-C, low-density lipoprotein cholesterol; ApoA1, apolipoprotein A1; ApoB, apolipoprotein B. Data are presented as mean ± standard deviation (SD) for continuous variables and number (percentage) for categorical variables. *p* values were calculated using Student’s t-test or Mann–Whitney U test for continuous variables and the chi-square test or Fisher’s exact test for categorical variables. *p* values represent comparisons between each MMD subgroup (ischemic or hemorrhagic) and healthy controls. *p < 0.05 was considered statistically significant.

The serum concentrations of leucine pathway-related metabolites are illustrated in Figure 2. MMD patients exhibited significantly higher serum leucine levels than HCs [217.91 (186.70–245.32) vs. 188.05 (166.09–217.76) μmol/L; *P* < 0.001]. Conversely, the levels of N-isovalerylglycine (N-IVA) were significantly lower in the MMD group [1.00 (0.96–1.05) vs. 1.03 (0.98–1.08) μmol/L; *p* = 0.004]. No significant difference in glycine levels was observed between the MMD and control groups (*p* = 0.482). Overall, the results indicate a distinct metabolic profile associated with alterations in the leucine pathway among patients with MMD. To address the substantial imbalance in group size and the possibility of confounding by cardiovascular risk-factor burden between the MMD and control groups, we performed two sensitivity analyses (Supplementary Table S4). First, propensity-score matching on age, sex, and BMI (1:3 caliper matching) yielded a matched sample of 218 MMD patients and 74 controls with markedly improved covariate balance; in this matched sample, the leucine association with MMD remained robust (Model 1-adjusted OR = 1.018, 95% CI 1.010–1.026, *p* < 0.001; cluster-robust standard errors accounting for the matched-set structure), whereas the N-IVA association was attenuated and no longer statistically significant (per 1-SD increase, OR = 0.824, 95% CI 0.598–1.134, *p* = 0.235). Second, restricting the MMD group to the 142 patients without any of five conventional vascular risk factors (hypertension, diabetes, hyperlipidemia, smoking, alcohol use) and comparing them with the 84 controls yielded a similar pattern: the leucine association remained significant after adjustment (OR = 1.012, 95% CI 1.004–1.021, *p* = 0.004), while the N-IVA association was directionally consistent but not statistically significant (per 1-SD increase, OR = 0.803, 95% CI 0.587–1.098, *p* = 0.170). These findings indicate that the leucine–MMD association is robust to case–control imbalance and cardiovascular risk-factor confounding, whereas conclusions regarding N-IVA should be interpreted more cautiously.

**Figure 2:**
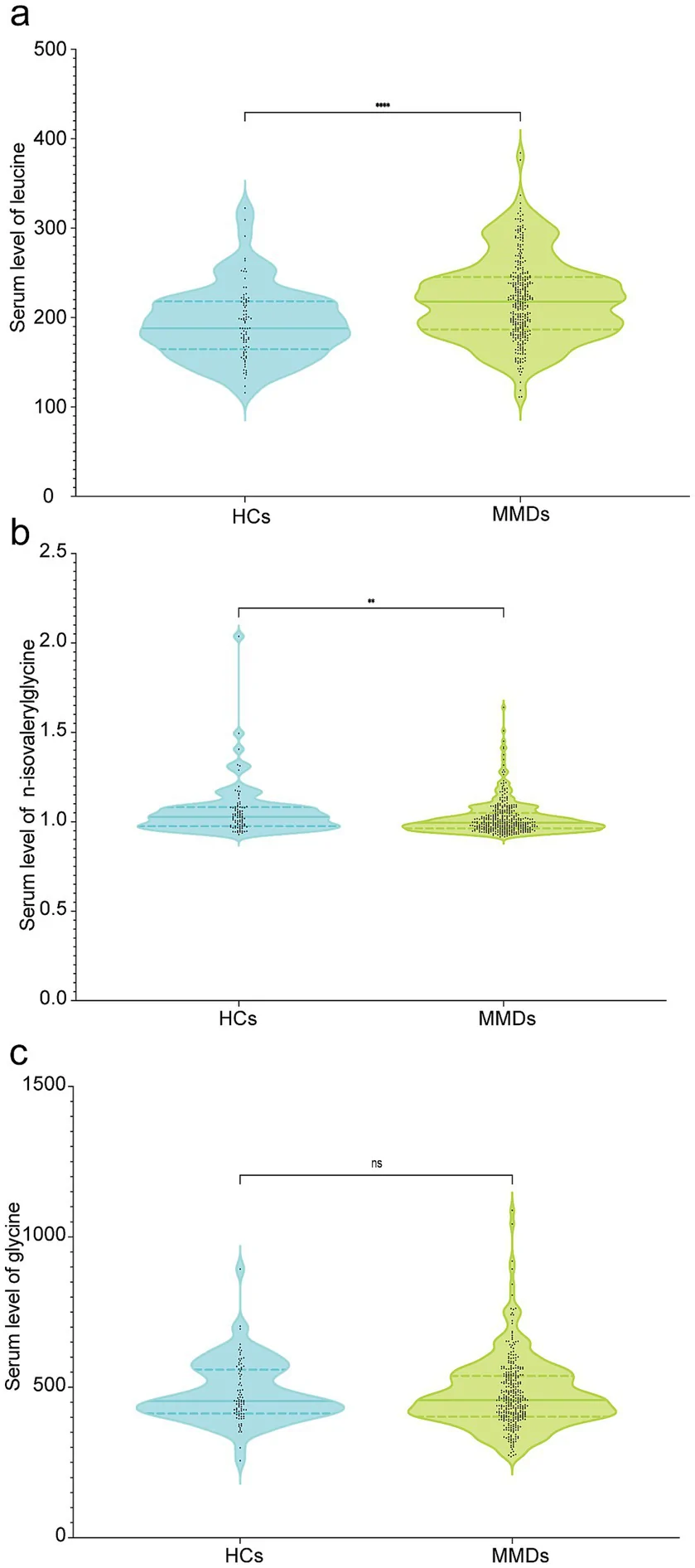
Serum concentrations of leucine pathway-related metabolites in patients with moyamoya disease (MMD) and healthy controls (HCs). Serum levels are shown for MMD patients (*n* = 337) versus healthy controls (*n* = 84): **(a)** Leucine, **(b)** N-isovalerylglycine (N-IVA), and **(c)** Glycine. Data are presented as median (IQR). Between-group comparisons were performed using the Mann–Whitney U test. *p < 0.05.

### 3.2 Associations of leucine-pathway metabolites with MMD and clinical phenotypes

To investigate the independent relationships between metabolites linked to the leucine pathway and the likelihood of MMD, multivariable logistic regression modeling was carried out (Tables 3–5). When considered as continuous measures, elevated serum leucine concentrations correlated with higher odds of MMD following comprehensive adjustment for established risk factors—such as blood pressure, lipid profiles, and homocysteine (Hcy)—as detailed in Model 2 (OR = 1.01, 95% CI: 1.00–1.02, *p* = 0.010). Conversely, increased levels of N-isovalerylglycine (N-IVA) were associated with lower odds of MMD (Model 2, per 1-SD increase, OR = 0.74, 95% CI: 0.55–1.00, *p* = 0.049). Glycine concentrations showed no statistically significant independent association with MMD (*p* = 0.368). In subgroup examinations, the inverse association of N-IVA with MMD persisted among individuals with the hemorrhagic MMD subtype, where those in the top tertile showed lower odds of MMD relative to those in the bottom tertile (Model 2, OR = 0.50, 95% CI: 0.25–0.98, *p* = 0.045).

**Table 3:** Association of leucine with moyamoya disease and its phenotypes.

Leucine, μmol/L	No. of events, *n* (%)	Crude model		Model1		Model2	
		OR (95% CI)	*p* value	OR (95% CI)	*p* value	OR (95% CI)	*p* value
Moyamoya overall							
Continuous	337 (80%)	1.01 (1.01, 1.02)	<0.001*	1.01 (1.01, 1.02)	<0.001*	1.01 (1.00, 1.02)	0.010*
Tertiles							
T1 (<197.83)	98 (69.5)	ref.		ref.		ref.	
T2 (197.83 ∼ 233.15)	111 (79.29)	1.68 (0.98, 2.91)	0.062	1.76 (0.96, 3.27)	0.068	1.15 (0.54, 2.43)	0.723
T3 (>233.15)	128 (91.43)	4.68 (2.41, 9.72)	<0.001*	4.63 (2.11, 10.76)	<0.001*	3.58 (1.34, 10.23)	0.013*
P value for trend			<0.001*		<0.001*		0.022*
Ischemic							
Continuous	249 (59.1%)	1.01 (1.01, 1.02)	<0.001*	1.01 (1.00, 1.01)	0.005*	1.00 (1.00, 1.01)	0.320
Tertiles							
T1 (<202.48)	67 (47.52)	ref.		ref.		ref.	
T2 (202.48 ∼ 236.43)	82 (58.57)	1.56 (0.98, 2.51)	0.064	1.42 (0.85, 2.37)	0.183	1.07 (0.58, 1.97)	0.816
T3 (>236.43)	100 (71.43)	2.76 (1.69, 4.55)	<0.001*	2.04 (1.15, 3.66)	0.015*	1.24 (0.62, 2.51)	0.539
P value for trend			<0.001*		0.015*		0.543
Hemorrhagic							
Continuous	88 (20.9%)	1.00 (0.99, 1.00)	0.376	1.00 (0.99, 1.01)	0.786	1.00 (1.00, 1.01)	0.197
Tertiles							
T1 (<198.80)	31 (21.99)	ref.		ref.		ref.	
T2 (198.80 ∼ 235.12)	29 (20.71)	0.93 (0.52, 1.64)	0.795	1.06 (0.58, 1.93)	0.851	1.01 (0.52, 1.94)	0.980
T3 (>235.12)	28 (20)	0.89 (0.50, 1.58)	0.683	1.25 (0.64, 2.42)	0.514	1.89 (0.89, 4.07)	0.100
P value for trend			0.682		0.520		0.116

Association of leucine with moyamoya disease and phenotypes (Tertiles). Odds ratios (ORs) and 95% confidence intervals (CIs) were estimated using logistic regression models. Crude model was unadjusted. Model 1 was adjusted for age, sex, heart rate, systolic blood pressure (SBP), diastolic blood pressure (DBP), and body mass index (BMI). Model 2 was further adjusted for laboratory parameters, including WBC, NEUT, PLT, GGT, glucose, creatinine, uric acid, triglycerides, total cholesterol, HDL-C, LDL-C, apolipoprotein A1, apolipoprotein B, homocysteine, and eGFR. Continuous variables represent per unit increase. Tertiles were analyzed with the lowest tertile (T1) as the reference. P for trend was calculated by treating tertiles as an ordinal variable.*p < 0.05 was considered statistically significant.

**Table 4:** Association of N-isovalerylglycine with moyamoya disease and its phenotypes.

NIVA, μmol/L	No. of events, *n* (%)	Crude model		Model1		Model2	
		OR (95% CI)	*p* value	OR (95% CI)	*p* value	OR (95% CI)	*p* value
Moyamoya overall							
Continuous	337 (80%)	0.75 (0.60, 0.93)	0.009*	0.74 (0.58, 0.95)	0.017*	0.74 (0.55, 1.00)	0.049*
Tertiles							
T1 (<0.86)	120 (85.11)	ref.		ref.		ref.	
T2 (0.86 ∼ 1.39)	117 (83.57)	0.89 (0.46, 1.70)	0.723	0.88 (0.44, 1.75)	0.706	0.79 (0.34, 1.82)	0.584
T3 (>1.39)	100 (71.43)	0.44 (0.24, 0.78)	0.006*	0.48 (0.25, 0.90)	0.025*	0.37 (0.16, 0.81)	0.014*
P value for trend			0.005*		0.020*		0.012*
Ischemic							
Continuous	249 (59.1%)	0.84 (0.68, 1.02)	0.077	0.86 (0.69, 1.07)	0.173	0.95 (0.74, 1.22)	0.699
Tertiles							
T1 (<0.86)	87 (61.7)	ref.		ref.		ref.	
T2 (0.86 ~ 1.35)	88 (62.86)	1.05 (0.65, 1.70)	0.842	1.08 (0.65, 1.82)	0.759	1.14 (0.63, 2.07)	0.661
T3 (>1.35)	74 (52.86)	0.70 (0.43, 1.12)	0.135	0.82 (0.49, 1.37)	0.451	1.01 (0.55, 1.85)	0.974
P value for trend			0.133		0.443		0.981
Hemorrhagic							
Continuous	88 (20.9%)	0.93 (0.72, 1.20)	0.550	0.90 (0.69, 1.17)	0.417	0.84 (0.63, 1.12)	0.229
Tertiles							
T1 (<0.83)	33 (23.4)	ref.		ref.		ref.	
T2 (0.83 ~ 1.52)	29 (20.71)	0.86 (0.48, 1.50)	0.587	0.82 (0.46, 1.47)	0.513	0.79 (0.42, 1.47)	0.455
T3 (>1.52)	26 (18.57)	0.75 (0.42, 1.33)	0.321	0.67 (0.36, 1.21)	0.187	0.50 (0.25, 0.98)	0.045*
P value for trend			0.320		0.187		0.045*

Association of N-Isovalerylglycine with Moyamoya disease and phenotypes (Tertiles). Odds ratios (ORs) and 95% confidence intervals (CIs) were estimated using logistic regression models. Crude model was unadjusted. Model 1 was adjusted for age, sex, heart rate, systolic blood pressure (SBP), diastolic blood pressure (DBP), and body mass index (BMI). Model 2 was further adjusted for laboratory parameters, including WBC, NEUT, PLT, GGT, glucose, creatinine, uric acid, triglycerides, total cholesterol, HDL-C, LDL-C, apolipoprotein A1, apolipoprotein B, homocysteine, and eGFR. For N-isovalerylglycine (N-IVA), whose raw concentrations span a narrow range (approximately 0.1–2.5 μmol/L), continuous-variable odds ratios are presented per 1-SD increase (SD = 0.109 μmol/L) to aid interpretability; the P value is mathematically identical to that for the per-unit (per 1 μmol/L) OR. Tertiles were analyzed with the lowest tertile (T1) as the reference. P for trend was calculated by treating tertiles as an ordinal variable.*p < 0.05 was considered statistically significant.

**Table 5:** Association of glycine with moyamoya disease and its phenotypes.

Glycine, μmol/L	No. of events, *n* (%)	Crude model		Model 1		Model 2	
		OR (95% CI)	*p* value	OR (95% CI)	*p* value	OR (95% CI)	*p* value
Moyamoya overall							
Continuous	337 (80%)	1.00 (1.00, 1.00)	0.729	1.00 (1.00, 1.00)	0.637	1.00 (1.00, 1.00)	0.368
Tertiles							
T1 (<233.72)	117 (82.98)	ref.		ref.		ref.	
T2(233.72 ∼ 282.68)	108 (77.14)	0.69 (0.38, 1.25)	0.222	0.78 (0.41, 1.46)	0.432	0.68 (0.31, 1.49)	0.341
T3 (>282.68)	112 (80)	0.82 (0.45, 1.50)	0.521	1.09 (0.57, 2.10)	0.793	1.22 (0.54, 2.75)	0.634
P value for trend			0.531		0.760		0.560
Ischemic							
Continuous	249 (59.1%)	1.00 (1.00, 1.00)	0.088	1.00 (1.00, 1.00)	0.427	1.00 (1.00, 1.00)	0.831
Tertiles							
T1 (237.95)	93 (65.96)	ref.		ref.		ref.	
T2 (237.95 ∼ 286.09)	77 (55)	0.63 (0.39, 1.02)	0.061	0.69 (0.41, 1.15)	0.159	0.67 (0.37, 1.23)	0.195
T3 (286.09)	79 (56.43)	0.67 (0.41, 1.08)	0.102	0.89 (0.53, 1.50)	0.666	0.97 (0.52, 1.79)	0.917
P value for trend			0.104		0.684		0.955
Hemorrhagic							
Continuous	88 (20.9%)	1.00 (1.00, 1.00)	0.085	1.00 (1.00, 1.00)	0.167	1.00 (1.00, 1.00)	0.189
Tertiles							
T1 (226.54)	24 (17.02)	ref.		ref.		ref.	
T2 (226.54 ∼ 277.67)	31 (22.14)	1.39 (0.77, 2.53)	0.280	1.36 (0.75, 2.50)	0.317	1.28 (0.67, 2.49)	0.455
T3 (277.67)	33 (23.57)	1.50 (0.84, 2.73)	0.174	1.33 (0.73, 2.44)	0.359	1.38 (0.72, 2.69)	0.333
P value for trend			0.178		0.371		0.339

Association of Glycine with Moyamoya disease and phenotypes (Tertiles). Odds ratios (ORs) and 95% confidence intervals (CIs) were estimated using logistic regression models. Crude model was unadjusted. Model 1 was adjusted for age, sex, heart rate, systolic blood pressure (SBP), diastolic blood pressure (DBP), and body mass index (BMI). Model 2 was further adjusted for laboratory parameters, including WBC, NEUT, PLT, GGT, glucose, creatinine, uric acid, triglycerides, total cholesterol, HDL-C, LDL-C, apolipoprotein A1, apolipoprotein B, homocysteine, and eGFR. Continuous variables represent per unit increase. Tertiles were analyzed with the lowest tertile (T1) as the reference. P for trend was calculated by treating tertiles as an ordinal variable. *p < 0.05 was considered statistically significant.

We further examined the distribution of clinical characteristics across tertiles of circulating metabolites (Supplementary Tables S1–S3). Across tertiles of leucine, higher levels tended to be associated with a greater proportion of ischemic presentation and a lower proportion of hemorrhagic presentation; however, this trend was not statistically significant and should not be interpreted as a robust finding (P for trend = 0.076). Patients in the highest leucine tertile also tended to have a higher proportion of mRS ≥ 2 (indicating at least slight disability) at admission, compared with those in the lowest tertile, although this difference likewise did not reach statistical significance (52.68% vs. 39.82%, P for trend = 0.053). However, no significant differences were observed in RNF213 p. R4810K mutation status, posterior involvement, or Suzuki stage distribution across leucine tertiles. For N-IVA and glycine, the distribution of clinical characteristics remained largely similar across tertiles. The proportions of RNF213 p. R4810K mutation, admission mRS score, posterior involvement, clinical presentation, and Suzuki stage did not differ significantly among the groups (all P for trend > 0.05). To directly compare metabolite levels between MMD phenotypes, rather than each subtype separately against healthy controls, we additionally compared all three metabolites between ischemic and hemorrhagic MMD directly (Supplementary Table S5). Unadjusted comparison showed modestly higher leucine levels in ischemic than hemorrhagic MMD (median 220.73 vs. 204.48 μmol/L, *p* = 0.023), while N-IVA did not differ significantly between subtypes (*p* = 0.985). After adjustment for age, sex, heart rate, SBP, DBP, and BMI, neither leucine (per 10 μmol/L, adjusted OR = 0.975, 95% CI 0.915–1.040, *p* = 0.442) nor N-IVA (per 1-SD, adjusted OR = 0.975, 95% CI 0.757–1.257, *p* = 0.848) differed significantly between the two phenotypes; glycine likewise showed no significant difference (per 50 μmol/L, adjusted OR = 1.066, 95% CI 0.958–1.186, *p* = 0.239), suggesting that phenotype-specific differences in these metabolites are modest once demographic and clinical factors are accounted for.

To assess the associations between metabolites in the leucine pathway and routine clinical parameters, Spearman rank correlation analysis was performed (Figure 3). Serum leucine levels were positively correlated with several metabolic indicators, including BMI, triglycerides (TG), and homocysteine (Hcy) (all *p* < 0.01), and showed modest positive correlations with liver function markers such as ALT, ALP, GGT, and cholinesterase (CHE). N-IVA demonstrated broadly similar correlation patterns with several metabolic and biochemical indices. In contrast, glycine showed inverse correlations with BMI and TG. Collectively, these findings suggest that alterations in leucine and N-IVA levels are associated with metabolic disturbances in patients with MMD.

**Figure 3:**
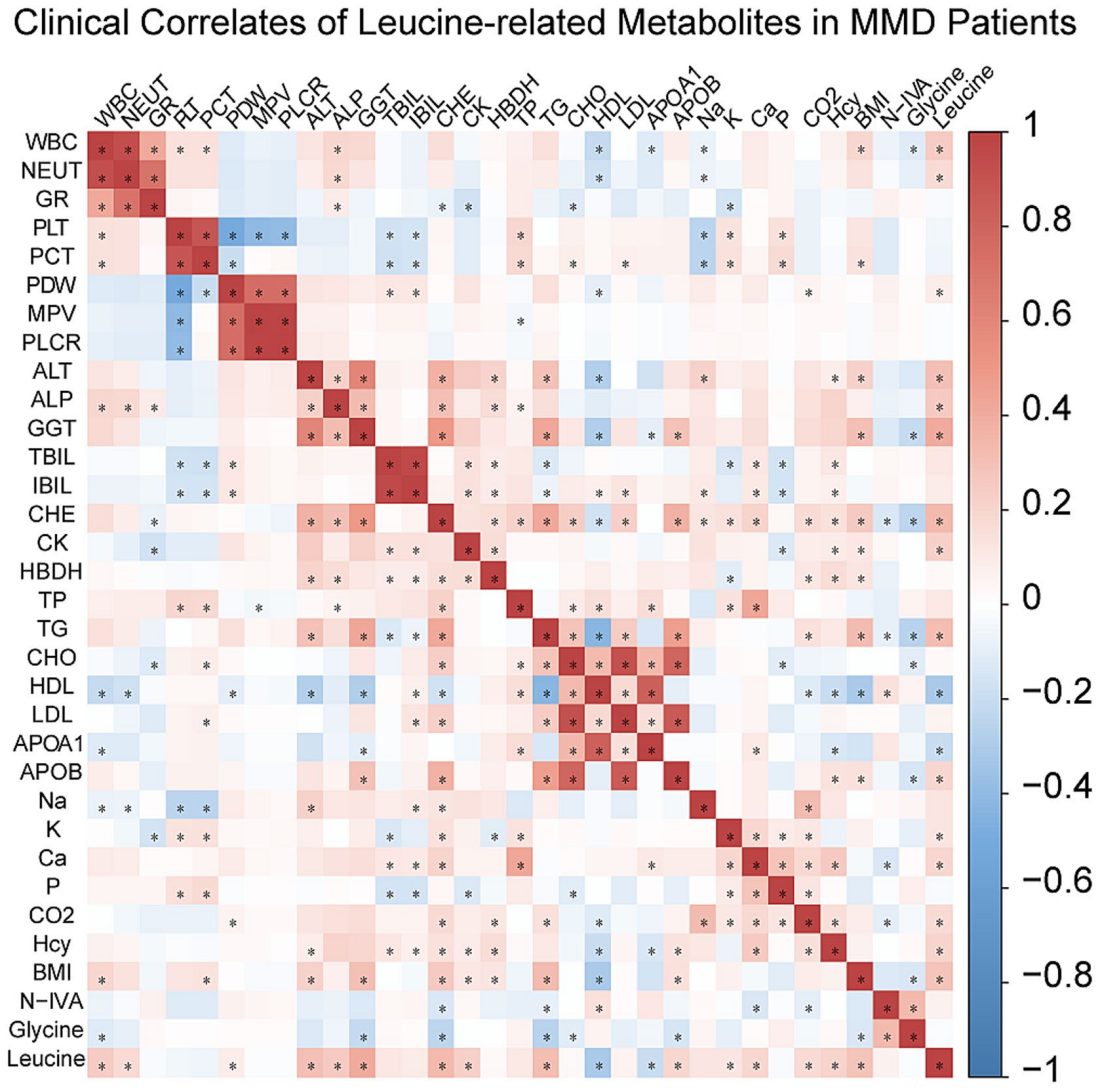
Spearman correlation analysis between leucine pathway metabolites and clinical parameters in patients with moyamoya disease (MMD). Spearman correlation coefficients between leucine, N-isovalerylglycine (N-IVA), glycine, and routine clinical/laboratory parameters among MMD patients (*n* = 337) are displayed in a heatmap, with red indicating positive correlations and blue indicating negative correlations. Color intensity reflects the magnitude of the correlation. Only statistically significant correlations are shown (*p < 0.05).

### 3.3 Incremental predictive value of leucine-pathway metabolites

To evaluate the discriminatory performance and clinical utility of leucine pathway-related metabolites, receiver operating characteristic (ROC) curve analyses were performed (Figure 4; Table 6). For individual metabolites, leucine demonstrated the most robust discriminative ability, with its AUC increasing from 0.670 (95% CI: 0.607–0.733) in the crude model to 0.886 (95% CI: 0.848–0.924) in the multivariable-adjusted model (Model 2). Similarly, the AUCs of models containing glycine or N-IVA together with the full Model 2 covariate set were 0.881 (95% CI: 0.842–0.921) and 0.883 (95% CI: 0.845–0.922), respectively; however, this reflects the strong discriminative performance of the Model 2 covariates themselves rather than an independent contribution of glycine or N-IVA, as neither metabolite’s addition to the basic model achieved a statistically significant categorical NRI or IDI individually (see below).

**Figure 4:**
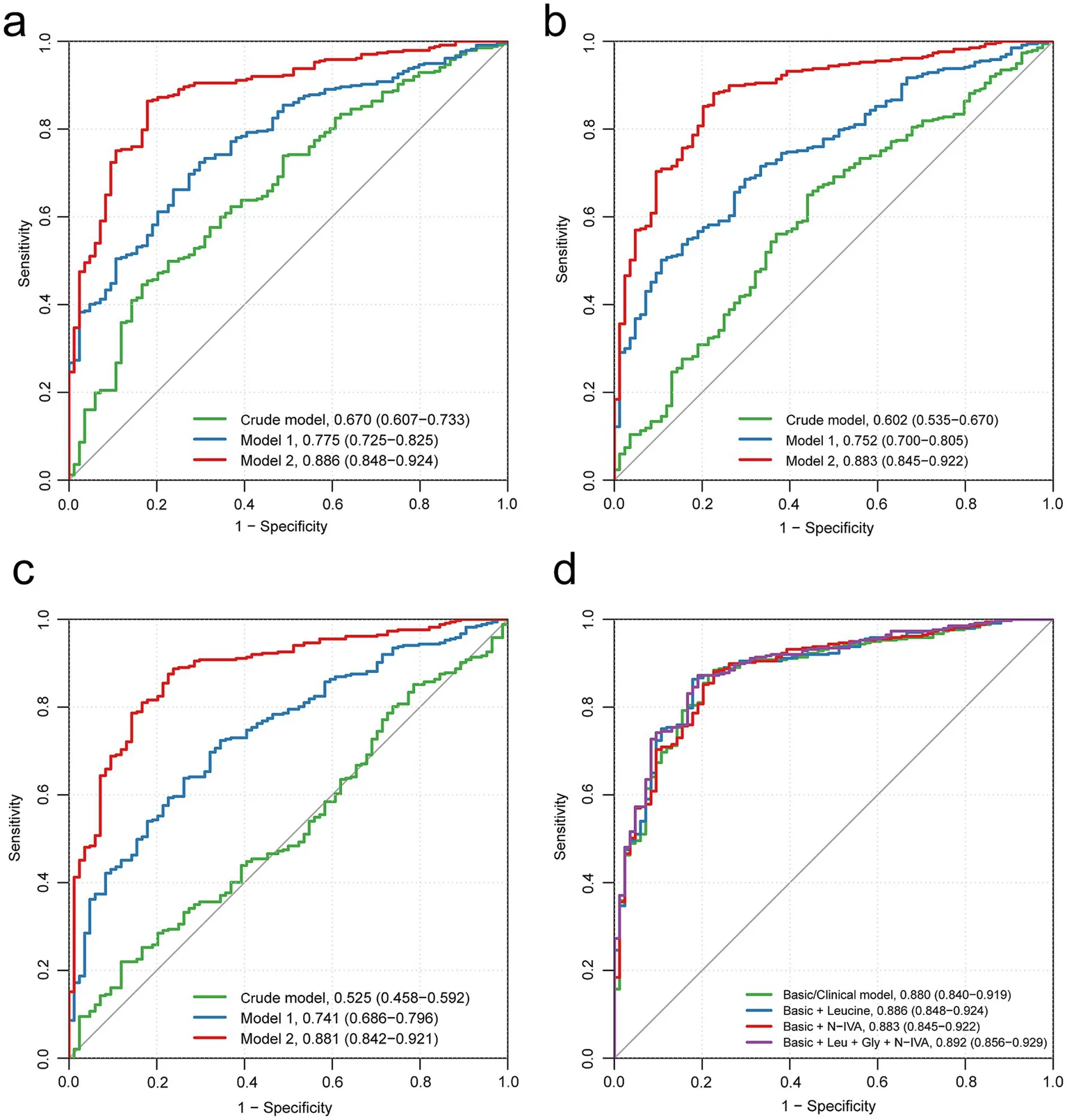
Incremental predictive value of leucine pathway metabolites for moyamoya disease (MMD) and its subtypes. Receiver operating characteristic (ROC) curves for **(a)** leucine, **(b)** N-isovalerylglycine (N-IVA), and **(c)** glycine, each shown for the crude (unadjusted) model, Model 1 (adjusted for age, sex, heart rate, SBP, DBP, and BMI), and Model 2 (Model 1 covariates plus WBC, neutrophil count, platelet count, GGT, fasting glucose, creatinine, uric acid, triglycerides, total cholesterol, HDL-C, LDL-C, apolipoprotein A1, apolipoprotein B, homocysteine, and eGFR), illustrating the gain in discriminative performance for each metabolite after adjustment. **(d)** ROC curves comparing the basic clinical model (i.e., the Model 2 covariates above, without any metabolite) with models additionally incorporating leucine, N-IVA, or all three metabolites combined, illustrating that the incremental gain in discrimination from adding metabolites to the basic clinical model is comparatively modest. The area under the curve (AUC) with 95% confidence intervals is reported for each curve. Net reclassification improvement (NRI) and integrated discrimination improvement (IDI), including ischemic- and hemorrhagic-subtype analyses (reported numerically rather than as separate ROC panels), are summarized in Table 6. AUC, area under the curve; NRI, net reclassification improvement; IDI, integrated discrimination improvement.

**Table 6:** Incremental predictive value of leucine pathway metabolites for moyamoya disease and its phenotypes.

Model	AUC (95% CI)	NRI		IDI	
		Categorical NRI (95% CI)	*p* value	IDI (95% CI)	*p* value
MMD overall					
Basic model	0.880 (0.840–0.919)	Ref.		Ref.	
Basic + Leucine	0.886 (0.848–0.924)	0.140 (0.032–0.238)	0.007*	0.017 (−0.000–0.035)	0.051
Basic + Glycine	0.881 (0.842–0.921)	0.015 (−0.054–0.091)	0.688	0.003 (−0.003–0.009)	0.355
Basic + N-IVA	0.883 (0.845–0.922)	0.042 (−0.036–0.116)	0.289	0.009 (−0.003–0.023)	0.148
Basic + Leu + Gly + N-IVA	0.892 (0.856–0.929)	0.193 (0.086–0.300)	<0.001*	0.032 (0.007–0.057)	0.011*
Ischemic MMD					
Basic model	0.825 (0.786–0.865)	Ref.		Ref.	
Basic + Leucine	0.826 (0.787–0.866)	0.035 (−0.022–0.092)	0.236	0.002 (−0.002–0.007)	0.291
Basic + Glycine	0.825 (0.785–0.865)	−0.018 (−0.044–0.010)	0.196	−0.000 (−0.001–0.001)	0.879
Basic + N-IVA	0.826 (0.786–0.865)	−0.004 (−0.035–0.027)	0.798	−0.000 (−0.002–0.002)	0.844
Basic + Leu + Gly + N-IVA	0.826 (0.787–0.866)	0.025 (−0.031–0.084)	0.386	0.002 (−0.003–0.007)	0.347
Hemorrhagic MMD					
Basic model	0.720 (0.665–0.776)	Ref.		Ref.	
Basic + Leucine	0.729 (0.675–0.783)	0.099 (0.002–0.210)	0.055	0.003 (−0.004–0.011)	0.403
Basic + Glycine	0.729 (0.675–0.784)	0.072 (−0.020–0.181)	0.167	0.003 (−0.003–0.011)	0.392
Basic + N-IVA	0.726 (0.670–0.782)	0.004 (−0.079–0.088)	0.934	0.006 (0.001–0.012)	0.024*
Basic + Leu + Gly + N-IVA	0.745 (0.691–0.800)	0.103 (−0.017–0.222)	0.092	0.019 (0.005–0.033)	0.010*

AUC, area under the curve; CI, confidence interval; NRI, net reclassification improvement; IDI, integrated discrimination improvement; N-IVA, N-isovalerylglycine. The basic model corresponds to Model 2 in logistic regression analyses and includes age, sex, heart rate, systolic blood pressure (SBP), diastolic blood pressure (DBP), body mass index (BMI), and laboratory parameters. Receiver operating characteristic (ROC) curve analysis was used to evaluate model discrimination, and the AUC with 95% confidence interval (CI) was calculated. Categorical NRI and IDI were used to assess the incremental predictive value of adding metabolites to the basic model. Risk categories for the categorical NRI were defined using tertiles of the predicted probability from the basic model (or quartiles/quintiles as a fallback if any tertile-based bin contained fewer than 10 observations, using the R package nricens), and IDI was estimated using bootstrap resampling (1,000 iterations, percentile 95% CI).*p < 0.05 was considered statistically significant.

The basic clinical model (Model 2), encompassing traditional cardiovascular risk factors and systemic laboratory indices, achieved a strong AUC of 0.880 (95% CI: 0.840–0.919). The integration of individual metabolites into this clinical model yielded incremental improvements in diagnostic accuracy. Notably, the combined incorporation of leucine, glycine, and N-IVA further augmented the discriminatory power, achieving a maximal AUC of 0.892 (95% CI: 0.856–0.929). The combined addition of leucine, glycine, and N-IVA resulted in a modest increase in AUC from 0.880 to 0.892. This finding suggests limited incremental discriminative value and requires validation in independent cohorts.

To further assess the clinical relevance of these biomarkers, we evaluated risk reclassification through the Net Reclassification Index (NRI) and Integrated Discrimination Improvement (IDI), as summarized in Table 6. Incorporating leucine alone led to a statistically significant improvement in MMD risk reclassification, achieving a categorical NRI of 0.140 (95% CI: 0.032–0.238; *p* = 0.007). Notably, the integrated metabolic panel—comprising leucine, glycine, and N-IVA—delivered even stronger predictive gains, reflected in a significant categorical NRI of 0.193 (95% CI: 0.086–0.300; *p* < 0.001) and a significant IDI of 0.032 (95% CI: 0.007–0.057; *p* = 0.011). Collectively, these metrics indicate that the leucine-associated metabolic profile meaningfully enhances MMD risk stratification beyond standard clinical predictors. This nested-model comparison—the basic clinical model with versus without leucine and N-IVA—directly addresses whether these metabolites provide independent diagnostic value beyond conventional clinical risk factors, as requested.

In phenotype-specific analyses, the incremental predictive value was particularly noteworthy for the hemorrhagic MMD subtype. While the improvement in the ischemic subtype was relatively modest, the combined model significantly enhanced the detection of the hemorrhagic phenotype, increasing the AUC from 0.720 to 0.745. This improvement was supported by a significant IDI of 0.019 (95% CI: 0.005–0.033; *p* = 0.010), suggesting that these metabolites may serve as sensitive indicators for identifying patients at risk for hemorrhagic presentations.

## 4 Discussion

In this case–control study, we comprehensively evaluated circulating leucine pathway metabolites and demonstrated that elevated serum leucine levels were independently correlated with a higher odds of MMD, exhibiting a clear dose–response relationship across tertiles. In contrast, N-isovalerylglycine (N-IVA), a downstream metabolite of the leucine shunt pathway, showed a significant inverse association with MMD, particularly in the hemorrhagic subtype. These associations remained robust after multivariable adjustment for traditional vascular risk factors and inflammatory markers. Moreover, the addition of leucine pathway metabolites to a conventional clinical model significantly improved risk discrimination and reclassification, as evidenced by incremental NRI and IDI. Taken together, these findings suggest that alterations in leucine pathway metabolism are associated with MMD. This association provides a novel metabolic perspective that complements the established understanding of genetic and vascular mechanisms in the disease.

Leucine is an essential branched-chain amino acid (BCAA) that serves not only as a fundamental building block for protein synthesis but also as a critical metabolic regulator of glucose homeostasis, lipid metabolism, and cellular growth (20, 21). Beyond its systemic metabolic roles, leucine acts as a potent nutrient-sensing signal that modulates vascular cell function (8). The observed elevation of circulating leucine in our study population may be associated with this vascular remodeling through several interrelated mechanisms. Specifically, leucine availability is closely coupled to the activation of mTORC1 signaling pathway (20). This process can be further potentiated by leucine-dependent production of acetyl-CoA, which facilitates protein acetylation and reinforces mTORC1 activity, suggesting a plausible metabolic–epigenetic interface linking systemic amino-acid status to vascular signaling (22). Importantly, dysregulated mTOR signaling has been implicated in cerebrovascular pathophysiology, including post-ischemic injury and endothelial-driven vascular malformations (23, 24). In the vascular compartment, leucine-driven mTORC1 activation impairs endothelium-dependent relaxation and engages oxidative stress–related signaling (25), thereby facilitating endothelial dysfunction. In parallel, endothelial-to-mesenchymal transition (EndMT)—a process in which endothelial cells acquire mesenchymal features—has been proposed as a potential process involved in vascular remodeling in MMD, based primarily on integrated public transcriptomic and machine-learning analyses with limited *in vitro* validation (26). Complementing these bioinformatic findings, an *in vitro* study showed that plasma-derived exosomes from patients with MMD induced EndMT-like phenotypic changes in cerebrovascular endothelial cells (27). However, direct evidence establishing EndMT as a driver of neointimal formation in human MMD lesions remains limited. Taken together, these observations raise the possibility that elevated leucine levels may be associated with mTORC1-related oxidative and phenotypic remodeling programs, which could contribute to the obstructive vascular changes characteristic of MMD. Further mechanistic studies are warranted to determine whether leucine directly modulates these signaling pathways in this disease context.

In contrast to the elevated leucine levels, we observed significantly reduced serum N-isovalerylglycine (N-IVA) levels in MMD patients. As N-IVA is generated through the glycine conjugation of leucine-derived acyl-CoA intermediates, it represents an alternative route for the detoxification and disposal of leucine-derived acyl-CoA intermediates. Experimental studies of hepatic xenobiotic metabolism suggest that mitochondrial ATP availability may influence glycine conjugation capacity (28, 29). Whether a comparable relationship exists between circulating N-IVA levels and mitochondrial function in MMD remains unknown and should be regarded as a hypothesis requiring experimental validation. The observed dissociation in our study population—pathological leucine accumulation with concomitantly reduced N-IVA—may be consistent with altered leucine-related metabolism; however, as we did not directly measure canonical leucine degradation intermediates (e.g., acetoacetate) or directly assess metabolic flux or mitochondrial function, this proposed mechanism remains hypothesis-generating rather than directly demonstrated. Dysregulated branched-chain amino acid (BCAA) metabolism has been widely implicated in mitochondrial dysfunction and heightened cellular stress across various metabolic disorders (30). Consistent with this, our correlation analysis revealed positive associations between leucine and systemic inflammatory markers as well as homocysteine, both of which are known to promote oxidative stress and endothelial dysfunction (8). Notably, N-IVA exhibited a significant inverse association specifically with the hemorrhagic phenotype of MMD. While the precise mechanism warrants further elucidation, this pattern may be consistent with phenotype-related differences in leucine-related metabolism; however, this hypothesis was not directly tested, and endothelial function was not assessed in this study. This is particularly relevant given that plasma-derived exosomes from patients with hemorrhagic MMD have been reported to induce mitochondrial dysfunction and vascular fragility in cerebrovascular endothelial cells (31). Collectively, the coexistence of elevated leucine and reduced N-IVA may reflect altered leucine-related metabolism in MMD. A potential relationship with impaired mitochondrial buffering remains speculative and requires direct experimental validation. In a direct head-to-head comparison between ischemic and hemorrhagic MMD (Supplementary Table S5), unadjusted leucine levels were modestly higher in the ischemic subtype, but this difference did not persist after adjustment for age, sex, heart rate, SBP, DBP, and BMI, and N-IVA did not differ between phenotypes at all. This suggests that phenotype-specific differences in leucine and N-IVA levels are otherwise modest once demographic and clinical factors are accounted for, and that the hemorrhagic-specific N-IVA association described above more directly reflects each phenotype’s relationship to healthy controls than a pronounced difference between the two MMD phenotypes themselves.

Regarding glycine, although no independent association with MMD was observed in multivariable models, higher circulating glycine levels consistently correlated with more favorable cardiometabolic profiles, including lower BMI and triglycerides. In population-based studies, circulating glycine is often considered a marker of metabolic health and is inversely associated with obesity and dyslipidemia (32, 33). Beyond serving as a substrate for N-IVA conjugation, glycine participates in redox regulation and anti-inflammatory processes (34, 35). However, our findings suggest that glycine may not be a direct determinant of MMD susceptibility. Instead, it likely reflects the broader cardiometabolic milieu accompanying systemic vascular remodeling. Collectively, while glycine was not independently associated with MMD, its association with favorable metabolic parameters underscores the importance of the systemic metabolic context in modulating vascular vulnerability.

This study has several limitations. Initially, the inherent constraints of a cross-sectional framework preclude the determination of causality. Future longitudinal cohorts are indispensable to clarify the chronological link between dysregulated leucine metabolism and the clinical advancement of MMD. Second, although multiple clinical confounders were considered, the possibility of residual confounding remains. Third, mechanistic interpretations were inferred from circulating metabolite profiles rather than direct cellular or molecular experiments. In addition, serum metabolite levels may not fully reflect the metabolic state of the local cerebrovascular microenvironment. Given the unique anatomical and hemodynamic characteristics of intracranial arteries, as well as potential blood–brain barrier influences, systemic metabolic alterations may not directly correspond to metabolic processes within affected vascular lesions. Future investigations integrating metabolomics with longitudinal follow-up, functional vascular studies, tissue-level analyses, and genetic stratification may further elucidate the biological relevance of leucine pathway metabolism in MMD. Fourth, although the control group was selected to represent a low-risk-factor reference population without vascular comorbidities—a design consistent with our group’s related studies in this same prospectively enrolled population (16, 19)—this approach could not fully rule out confounding by cardiovascular risk-factor burden. To address this, we performed propensity-score matching (age, sex, BMI) and a sensitivity analysis restricting the MMD group to risk-factor-free patients; the leucine association with MMD remained robust in both analyses, whereas the N-IVA association was attenuated under reduced statistical power, and this should be considered when interpreting the N-IVA findings. Fifth, the current targeted panel was restricted to leucine, glycine, and N-IVA; isoleucine and valine, which our group has characterized separately in this same population (16), were not included, and canonical leucine degradation intermediates (e.g., acetoacetate) were not directly measured, so the proposed shunt-pathway mechanism remains hypothesis-generating. Finally, all models developed in this study are cross-sectional diagnostic (discriminative) models distinguishing prevalent MMD cases from controls; no prognostic model predicting future clinical outcomes was developed, which represents an important direction for future longitudinal research.

## 5 Conclusion

In conclusion, this study identifies a distinct leucine-related metabolic signature associated with MMD. Elevated leucine showed a robust, independent association with higher odds of MMD across multiple sensitivity analyses, while its shunt metabolite N-isovalerylglycine showed a directionally consistent inverse association, particularly in hemorrhagic cases, that was more sensitive to sample size and should be interpreted cautiously. Together, these metabolites provided a modest but statistically significant improvement in MMD risk reclassification beyond conventional clinical factors, offering a new metabolic perspective that may inform future precision screening strategies for MMD, pending validation in independent and longitudinal cohorts.

## Data Availability

The raw data supporting the conclusions of this article will be made available by the authors, without undue reservation.
