# Supplementary material for: Association of leucine pathway metabolites with moyamoya disease and its clinical phenotypes
Source: Front Neurol. 2026 Oct 8;17:1875390. doi: 10.3389/fneur.2026.1875390 (PMC13686879; doi:10.3389/fneur.2026.1875390)
Supplement: Supplementary file 1 [file Data_sheet_1.pdf]

Supplementary Material

Supplementary Table S1. Clinical characteristics of MMD patients according to tertiles of plasma leucine.

Clinical characteristics, n (%)	T1 (Low)	T2 (Mid)	T3 (High)	P for trend
RNF213 p.R4810K	25 (22.12)	19 (16.96)	16 (14.29)	0.126
mRS score ≥ 2 at admission	45 (39.82)	46 (41.07)	59 (52.68)	0.053
Posterior involvement	18 (15.93)	18 (16.07)	11 (9.82)	0.189
Clinical manifestations				0.076
Ischemic	78 (69.03)	82 (73.21)	89 (79.46)	
Hemorrhagic	35 (30.97)	30 (26.79)	23 (20.54)	
Suzuki stage				0.344
0–2	30 (26.55)	31 (27.68)	36 (32.14)	
3–4	59 (52.21)	51 (45.54)	56 (50.00)	
5–6	24 (21.24)	30 (26.79)	20 (17.86)	

MMD, moyamoya disease; mRS, modified Rankin Scale; T1–T3, tertile 1 (lowest) to tertile 3 (highest) of leucine concentration. P for trend was calculated by treating tertile group as an ordinal variable.

Supplementary Table S2. Clinical characteristics of MMD patients according to tertiles of plasma glycine.

Clinical characteristics, n (%)	T1 (Low)	T2 (Mid)	T3 (High)	P for trend
RNF213 p.R4810K	16 (14.16)	19 (16.96)	25 (22.32)	0.111
mRS score ≥ 2 at admission	51 (45.13)	52 (46.43)	47 (41.96)	0.634
Posterior involvement	11 (9.73)	20 (17.86)	16 (14.29)	0.324
Clinical manifestations				0.064
Ischemic	92 (81.42)	78 (69.64)	79 (70.54)	
Hemorrhagic	21 (18.58)	34 (30.36)	33 (29.46)	
Suzuki stage				0.782
0–2	35 (30.97)	29 (25.89)	33 (29.46)	
3–4	55 (48.67)	56 (50.00)	55 (49.11)	
5–6	23 (20.35)	27 (24.11)	24 (21.43)	

MMD, moyamoya disease; mRS, modified Rankin Scale; T1–T3, tertile 1 (lowest) to tertile 3 (highest) of glycine concentration. P for trend was calculated by treating tertile group as an ordinal variable.

Supplementary Table S3. Clinical characteristics of MMD patients according to tertiles of plasma N-isovalerylglycine (N-IVA).

Clinical characteristics, n (%)	T1 (Low)	T2 (Mid)	T3 (High)	P for trend
RNF213 p.R4810K	20 (17.70)	18 (16.07)	22 (19.64)	0.704
mRS score ≥ 2 at admission	48 (42.48)	51 (45.54)	51 (45.54)	0.644
Posterior involvement	17 (15.04)	13 (11.61)	17 (15.18)	0.979
Clinical manifestations				0.911
Ischemic	83 (73.45)	83 (74.11)	83 (74.11)	
Hemorrhagic	30 (26.55)	29 (25.89)	29 (25.89)	
Suzuki stage				0.109
0–2	36 (31.86)	35 (31.25)	26 (23.21)	
3–4	53 (46.90)	58 (51.79)	55 (49.11)	
5–6	24 (21.24)	19 (16.96)	31 (27.68)	

MMD, moyamoya disease; mRS, modified Rankin Scale; N-IVA, N-isovalerylglycine; T1–T3, tertile 1 (lowest) to tertile 3 (highest) of N-IVA concentration. P for trend was calculated by treating tertile group as an ordinal variable.

Supplementary Table S4. Sensitivity analyses addressing case-control imbalance and cardiovascular risk-factor confounding.

A. Propensity-score matching (age, sex, BMI; 1:3 caliper matching within 0.2 SD of the logit propensity score) — covariate balance before and after matching

	Before matching			After matching		
**Variable**	**HC (n=84)**	**MMD (n=337)**	**SMD**	**HC (n=74)**	**MMD (n=218)**	**SMD**
Age, years (mean ± SD)	39.9 ± 11.8	42.3 ± 10.5	0.214	41.1 ± 11.7	41.5 ± 10.7	0.032
Male sex, n (%)	34 (40.5)	140 (41.5)	0.022	32 (43.2)	89 (40.8)	−0.049
BMI, kg/m² (mean ± SD)	23.76 ± 3.37	25.49 ± 4.43	0.438	24.33 ± 3.14	24.31 ± 3.42	−0.007

B. Association of serum leucine, N-IVA, and glycine with MMD in the propensity-score-matched sample (n = 292: 218 MMD, 74 HC)

Metabolite	Model	OR (95% CI)	P value
**Leucine, per 1 μmol/L**	Crude	1.0137 (1.0068–1.0207)	<0.001
	Model 1-adjusted	1.0182 (1.0102–1.0261)	<0.001
**N-IVA, per 1-SD**	Crude	0.8214 (0.6401–1.0541)	0.122
	Model 1-adjusted	0.8238 (0.5983–1.1341)	0.235
**Glycine, per 1 μmol/L**	Crude	0.9998 (0.9975–1.0020)	0.844
	Model 1-adjusted	1.0005 (0.9976–1.0033)	0.751

C. Risk-factor-free sensitivity analysis: MMD patients without hypertension, diabetes, hyperlipidemia, smoking, or alcohol use, compared with healthy controls

Metabolite	Risk-factor-free MMD (n=142), median [IQR]	HC (n=84), median [IQR]	Crude OR (95% CI), P	Model 1-adjusted OR (95% CI), P
Leucine, per 1 μmol/L	195.96 [177.74–227.92]	188.05 [166.09–217.76]	1.0066 (0.9998–1.0135), 0.059	1.0124 (1.0038–1.0210), 0.004
N-IVA, per 1-SD	0.99 [0.96–1.06]	1.03 [0.98–1.08]	0.7710 (0.5810–1.0231), 0.071	0.8030 (0.5870–1.0984), 0.170
Glycine, per 1 μmol/L	470.75 [411.75–548.57]	454.41 [413.88–555.46]	1.0011 (0.9987–1.0034), 0.364	1.0014 (0.9988–1.0040), 0.288

SMD, standardized mean difference; BMI, body mass index; HC, healthy control; MMD, moyamoya disease; N-IVA, N-isovalerylglycine; OR, odds ratio; CI, confidence interval; IQR, interquartile range; SD, standard deviation. Model 1 was adjusted for age, sex, heart rate, systolic blood pressure, diastolic blood pressure, and BMI. In Panel B, because the 1:3 caliper matching without replacement induces within-matched-set correlation, standard errors were estimated using a cluster-robust (sandwich) variance, with each control and its matched case(s) treated as one cluster (74 clusters); Panel C compares independent, non-matched groups, so conventional model-based standard errors were used. Odds ratios for leucine and glycine are expressed per 1-μmol/L increase; because N-IVA has a narrow concentration range, its odds ratio is expressed per 1-SD increase (SD = 0.101 μmol/L in Panel B; 0.125 μmol/L in Panel C) for interpretability. P < 0.001 is reported as "<0.001"; other P values are reported to 3 decimal places.

Supplementary Table S5. Direct comparison of leucine, N-isovalerylglycine (N-IVA), and glycine between ischemic and hemorrhagic MMD phenotypes.

Metabolite	Ischemic MMD (n=249), median [IQR]	Hemorrhagic MMD (n=88), median [IQR]	Unadjusted P[a]	Adjusted OR (95% CI)[b]	Adjusted P
Leucine, per 10 µmol/L	220.73 [189.61–249.77]	204.48 [181.23–237.44]	0.023	0.9753 (0.9151–1.0395)	0.442
N-IVA, per 1-SD	0.99 [0.96–1.05]	1.00 [0.96–1.05]	0.985	0.9754 (0.7571–1.2568)	0.848
Glycine, per 50 µmol/L	454.16 [400.58–537.24]	466.33 [418.98–538.00]	0.090	1.0661 (0.9583–1.1860)	0.239

MMD, moyamoya disease; N-IVA, N-isovalerylglycine; IQR, interquartile range; OR, odds ratio; CI, confidence interval; SD, standard deviation. [a]Unadjusted between-phenotype comparison by the Mann–Whitney U test. [b]Adjusted OR represents the odds of the hemorrhagic (vs. ischemic) phenotype per the stated increment in metabolite concentration, adjusted for age, sex, heart rate, systolic blood pressure, diastolic blood pressure, and BMI (Model 1). Increments were chosen for clinical readability: leucine per 10 µmol/L, N-IVA per 1-SD (SD = 0.095 µmol/L in this sample), and glycine per 50 µmol/L.
